# Comprehensive Evaluation of the Immune Risk Phenotype in Successfully Treated HIV-Infected Individuals

**DOI:** 10.1371/journal.pone.0117039

**Published:** 2015-02-03

**Authors:** Patricia Ndumbi, Louise Gilbert, Christos M. Tsoukas

**Affiliations:** Immune Deficiency Treatment Centre, McGill University Health Centre, Montreal, QC, Canada; University of Montreal Hospital Research Center (CRCHUM), CANADA

## Abstract

**Background:**

Despite successful treatment and CD4+ T-cell recovery, HIV-infected individuals often experience a profound immune dysregulation characterized by a persistently low CD4:CD8 T-cell ratio. This residual immune dysregulation is reminiscent of the Immune Risk Phenotype (IRP) previously associated with morbidity and mortality in the uninfected elderly (>85 years). The IRP consists of laboratory markers that include: a low CD4:CD8 T-cell ratio, an expansion of CD8+CD28- T-cells and cytomegalovirus (CMV) seropositivity. Despite the significant overlap in immunological phenotypes between normal aging and HIV infection, the IRP has never been evaluated in HIV-infected individuals. In this pilot study we characterized immune changes associated with the IRP in a sample of successfully treated HIV-infected subjects.

**Methods:**

18 virologically suppressed HIV-infected subjects were categorized into 2 groups based on their IRP status; HIV+IRP+, (n = 8) and HIV+IRP-, (n = 10) and compared to 15 age-matched HIV uninfected IRP negative controls. All individuals were assessed for functional and phenotypic immune characteristics including: pro-inflammatory cytokine production, antigen-specific proliferation capacity, replicative senescence, T-cell differentiation and lymphocyte telomere length.

**Results:**

Compared to HIV-infected subjects without an IRP, HIV+IRP+ subjects exhibited a higher frequency of TNF-α-producing CD8+ T-cells (*p* = 0.05) and a reduced proportion of CD8+ naïve T-cells (*p* = 0.007). The IRP status was also associated with a marked up-regulation of the replicative senescence markers CD57 and KLGR1, on the surface of CD8+T-cells (*p* = 0.004). Finally, HIV+IRP+ individuals had a significantly shorter mean lymphocyte telomere length than their non-IRP counterparts (*p* = 0.03).

**Conclusions:**

Our findings suggest that, despite similar levels of treatment-mediated viral suppression, the phenotypic and functional immune characteristics of HIV+IRP+ individuals are distinct from those observed in non-IRP individuals. The IRP appears to identify a subset of treated HIV-infected individuals with a higher degree of immune senescence.

## Introduction

Although the hallmark of HIV infection is progressive CD4+ T-cell depletion, other impairments in immune phenotype occur, including an inversion of the CD4:CD8 T-cell ratio [1]. In the short term, effective antiretroviral therapy (ART) increases CD4+ T-cell counts [2]. However, this increase may not accurately reflect long-term immune recovery, as the majority of ART-treated individuals maintain a profound and persistent immune dysregulation as defined by an abnormally low CD4:CD8 T-cell ratio [3,4]. Our previous study on a sample of 6673 HIV-infected adults enrolled in the Canadian Observational Cohort (CANOC) revealed that less than ten percent of those treated achieved normalization of their CD4:CD8 T-cell ratio [5]. The abnormally low CD4:CD8 T-cell ratio and other residual immune alterations found in successfully treated HIV-infected individuals resemble the immune risk phenotype (IRP) previously identified in very elderly uninfected individuals [6,7]. Longitudinal geriatric studies have shown an association between the IRP and increased risk of mortality among octagerians [8,9]. The IRP consists of a combination of phenotypic and serologic parameters that include: a low CD4:CD8 T-cell ratio, an expansion of the CD8+CD28- T-cell subset and the presence of cytomegalovirus (CMV) seropositivity [10–12]. Elderly individuals with an IRP have increased susceptibility to infections, reactivation of latent pathogens and decreased responses to vaccination [13–15]; reflecting an age-related loss of T-cell function. The IRP thus defines the biological aging of the immune system, often referred to as immune senescence [16–18]. This phenotype has also been associated with markers of immune aging such as the depletion of naïve T-cells, the expansion of terminally differentiated memory T-cells, the expression of markers of replicative senescence such as CD57 and KLRG-1, as well as the production of pro-inflammatory cytokines [9,12,19,20]. The hallmark of cellular senescence is the shortening of telomeres at the end of chromosomes [21]. Chronic CMV infection contributes to telomere attrition in circulating T-cells [22]. However, telomere length has not yet been investigated in relation to the IRP. Considering the significant overlap in clinical and immunological phenotypes observed in the context of normal aging and HIV infection, we were interested in characterizing IRP-associated immune abnormalities in HIV-infected individuals. To our knowledge, an evaluation of the IRP that includes telomere changes has never been reported within the HIV population. Therefore, we assessed the IRP in a comprehensive fashion in successfully treated HIV-infected individuals, and investigated its relationship to phenotypic and functional markers of immune senescence.

## Materials and Methods

### Ethics statement

This research was approved by the institutional review board of the Montreal General Hospital (MGH). All participants provided written informed consent.

### Participants

The IRP was defined as the combination of a low CD4:CD8 ratio (<1), an expansion of CD8+CD28- T-cells of >50% of the peripheral blood lymphocytes and the presence of CMV specific IgG antibodies. Eighteen men chronically infected with HIV were studied. All met the following study entry criteria: (1) absence of active clinical manifestations (2) successful treatment with ART for at least 2 years (<50 copies HIV-1 RNA/ml plasma). Subjects were excluded from participation if they had: (1) use of immunomodulatory therapy (2) evidence of any acute infection, including active opportunistic infections, malignancy, or febrile illness. The 18 subjects were recruited and categorized based on their IRP status (IRP negative (-) = 8 subjects, IRP positive (+) = 10 subjects) and compared to 15 age-matched HIV uninfected healthy male controls that did not have an IRP and were negative for the presence of any immune deficiencies or autoimmune diseases.

All healthy controls and all but one of the HIV-infected subjects were Caucasian. The median (interquartile range [IQR]) age was 58 yrs (48–64), 78% were men who have sex with men (MSM) and 83% were on a regimen that included nucleosides. There were no significant differences in age, treatment regimen or risk group between controls, HIV+IRP+ and HIV+IRP- groups. Additional information on the subjects has been summarized in **Table 1**.

**Table 1:** Participants’ characteristics.

Participant	Status	Age	CMV	Anti-CMV IgG titers (AU/mL)	CD4:CD8	%CD4	CD4 Abs	%CD8	CD8 Abs	%CD8+CD28-
1	Controls	48	Positive	132.4	4.25	68	1428	16	336	43
2	Controls	49	Positive	>250	2.74	52	884	19	323	47
3	Controls	58	Negative	9.6	4.07	57	456	14	112	17
4	Controls	49	Negative	0	5.44	49	784	9	144	22
5	Controls	39	Negative	3	1.60	40	800	25	500	41
6	Controls	53	Negative	0	6.30	63	1260	10	200	31
7	Controls	43	Positive	27	1.70	39	663	23	391	47
8	Controls	58	Negative	0	2.18	48	912	22	418	43
9	Controls	52	Negative	0	1.92	48	576	25	300	33
10	Controls	68	Positive	>250	2.08	50	850	24	408	67
11	Controls	49	Negative	0	0.75	30	720	40	960	39
12	Controls	52	Positive	123	3.43	48	1100	14	321	35
13	Controls	53	Negative	0	2.84	54	918	19	323	28
14	Controls	61	Negative	0	2.09	46	782	22	374	17
15	Controls	67	Negative	1	3.73	41	1148	11	308	15
16	HIV-IRPneg	47	Positive	>250	1.23	38	960	31	1008	39
17	HIV-IRPneg	64	Negative	6.3	1.62	42	1350	26	729	37
18	HIV-IRPneg	46	Negative	3.1	0.38	21	396	56	1062	46
19	HIV-IRPneg	42	Negative	2.3	0.97	35	608	36	874	47
20	HIV-IRPneg	41	Negative	0	2.08	50	1428	24	700	39
21	HIV-IRPneg	58	Negative	0	1.20	42	546	35	616	49
22	HIV-IRPneg	64	Negative	4.6	1.38	36	1617	26	858	42
23	HIV-IRPneg	59	Positive	116.7	1.80	45	688	25	480	42
24	HIV-IRPpos	62	Positive	>250	0.33	19	425	57	1020	50
25	HIV-IRPpos	71	Positive	>250	0.79	26	1116	33	1209	84
26	HIV-IRPpos	51	Positive	>250	0.42	23	540	55	1431	72
27	HIV-IRPpos	60	Positive	>250	0.29	16	150	56	600	52
28	HIV-IRPpos	64	Positive	>250	0.66	29	754	44	1404	86
29	HIV-IRPpos	57	Positive	>250	0.64	25	870	39	1305	65
30	HIV-IRPpos	58	Positive	>250	0.61	25	340	41	460	56
31	HIV-IRPpos	49	Positive	>250	0.76	32	792	42	1080	51
32	HIV-IRPpos	48	Positive	>250	0.89	40	272	45	901	66
33	HIV-IRPpos	68	Positive	>250	0.39	14	196	36	546	71

### Laboratory methods

**CMV Serology**. CMV IgG levels were determined in subjects’ sera by using a commercial microparticle enzyme immunoassay (Abbott AxSYM; Abbott Laboratories). Briefly, 5 ml of whole blood was collected in a serum separator tube and the sample was centrifuged (3,000 rpm x 5 minutes). The collected serum specimens were assessed for CMV IgG positivity using the Abbott AxSYM System. All tests were performed and the results were interpreted according to the manufacturer’s instructions. Specimens containing a CMV IgG concentration >25 AU/mL were considered seropositive.

**Preparation of peripheral blood mononuclear cells (PBMC).** PBMC were isolated by density gradient centrifugation (Ficoll-Paque, Sigma-Aldrich) from whole blood obtained by venipuncture into tubes containing ACD anticoagulant. Proliferation assays were performed using fresh cells. Immune phenotype, intracellular cytokine detection and telomere measurement assays were performed at a later time point using PBMC that were cryopreserved in 10% DMSO (Sigma-Aldrich) with 90% fetal bovine serum (FBS, Wisent) and stored in liquid nitrogen.

**Proliferation assays**. A carboxyfluorescein succinimidyl ester (CFSE) dilution assay was used to measure the proliferative capacity of lymphocytes in response to antigen and mitogen stimulation. Fresh PBMC were labelled with 1 mM CFSE (Invitrogen, Molecular Probe) according to the manufacturer’s instructions. Cells were resuspended at a concentration of 10^6^/mL in RPMI 1640 medium supplemented with 15% human AB serum (Wisent), 2 mM L-glutamine (Wisent), 50 IU/ml penicillin (Wisent) and 50 μg/ml streptomycin (Wisent) (cRPMI-15). The cells were either left untreated (negative control) or stimulated with a panel of stimuli including: PHA (5 μg/ml, Sigma Aldrich), anti-CD3 monoclonal antibody (mAb) (1:1000, Research Diagnostics), anti-CD28 mAb (1:1000, Research Diagnostics), Pokeweed mitogen (5 μg/mL, Sigma Aldrich) and Tetanus toxoid (1LF/mL, Pasteur Merieux Connaught). After 7 days at 37C in a humidified 5% CO_2_ incubator, cells were washed twice in FACSflow buffer (BD Biosciences) and stained with anti-CD45 PerCP mAb. 20,000 events were acquired on a FACSCalibur flow cytometer (Becton Dickinson). Results were analyzed with the BD CellQuest software. BD Calibrite beads were used to set photomultuplier voltages and fluorescence compensation and to check instrument sensitivity before each experiment. The data obtained were corrected for background staining of unstimulated cells before statistical analysis.

**Flow cytometry analysis**. Cryopreserved PBMCs were thawed, washed in phosphate buffered saline (PBS) and stained with one of 2 mAb cocktails (Cocktail 1—Maturation panel: Live/Dead UV-Blue (Life Technologies), anti-CD45-PerCP-Cy5.5 (Ebioscience), anti-CD3-PE-CF594 (BD Biosciences), anti-CD4-PE (Ebioscience), anti-CD8-APC (Ebioscience), anti-CD45RA-PE-Cy7 (Ebioscience), anti-CCR7-BV-421 (Biolegend) and anti-CD28-FITC (EBioscience). Cocktail 2—Senescence panel: Live/Dead UV-Blue (Life Technologies), anti-CD3-PerCP-Cy5.5 (Ebioscience), anti-CD4-BV 421 (Biolegend), anti-CD8-APC (Ebioscience), anti-CD57-PE-CF594 (BD Biosciences) and anti-KLRG1-PE (Biolegend). 10^6^ PBMC were stained with each Ab panel for 30 minutes at RT and washed twice prior to acquisition. 200,000 events were acquired per condition with an LSRFortessa flow cytometer (BD Biosciences) using the Diva 4.1 software (BD Biosciences). CompBeads were used to set up voltage and compensation settings and fluorescence minus one (FMO) controls tubes were prepared for each run to establish gate settings between runs. Data analysis was performed using FlowJo software version 9.6 (TreeStar).

**Intracellular cytokine staining (ICS).** Cryopreserved PBMCs were thawed, washed twice in RPMI 1640 medium and resuspended in cRPMI-15. Cells were then stimulated for 24 hrs with 40 μL of staphylococcal enterotoxin B (SEB) (50μg/ml, Sigma) and 7 μL of a mAb cocktail to the co-stimulatory molecules CD28 and CD49d. After 2 hours of stimulation, intracellular protein transport was blocked by adding GolgiPlug (1 μL/mL, Becton Dickinson). Cells were then washed, fixed and permeabilized using the Fix and Perm kit (Becton Dickinson) according to manufacturer directions. PBMC were subsequently stained with 3 different mAb cocktails including Abs to cell surface markers CD3-PerCP, CD4-APC and CD28-FITC—all from EBiosciences) and a PE-conjugated Ab to one of the following intracellular cytokines: IFN-γ, TNF-α or IL-2. The data obtained were corrected for background staining of unstimulated cells before statistical analysis.

**Telomere Length Analysis**. The absolute telomere length (aTL) of genomic DNA from peripheral blood lymphocytes was determined by real-time quantitative polymerase chain reaction (qPCR) using a modification of a previously published protocol for measurement of relative telomere length [23,24]. Briefly, DNA was extracted from cryopreserved and thawed PBMC using a QIAamp DNA mini kit (Qiagen). The concentration and purity of DNA were assessed by UV spectroscopy (Nanodrop, Thermo Fisher Scientific Inc.). All DNA samples were diluted to a fixed concentration of 5 ng/μl. PCR reactions were performed using the LightCycler 480 real-time PCR detection system (Roche). PCR reactions were performed in triplicates using equal amounts of DNA (20 ng). For each reaction, 2 standard curves were made using serial dilutions of known amounts of telomeric (T) and a reference control gene 36B4 single copy gene (S) DNA oligonucleotide (Integrated DNA Technologies). Utilizing primers (Integrated DNA Technologies) specific for telomeric hexamer repeats and 36B4 SCG, the copy number of T DNA was compared to that of S DNA in order to generate a T/S ratio indicative of the relative telomere length for each DNA sample. Absolute telomere length was determined by dividing the telomere kilobase (kb) per reaction value by the number of diploid genome copies (estimated from the SCG 36B4 standard curve) to generate a total telomeric length in kb per diploid genome.

### Statistical analysis

Statistical analyses and graphical presentations were performed using Graphpad Prism version 4. The Mann-Whitney U-tests were used for comparisons between independent groups. *p*-values <0.05 were considered significant.

## Results

### 1. Relationship between the IRP and the production of inflammatory cytokines

**Fig 1:**
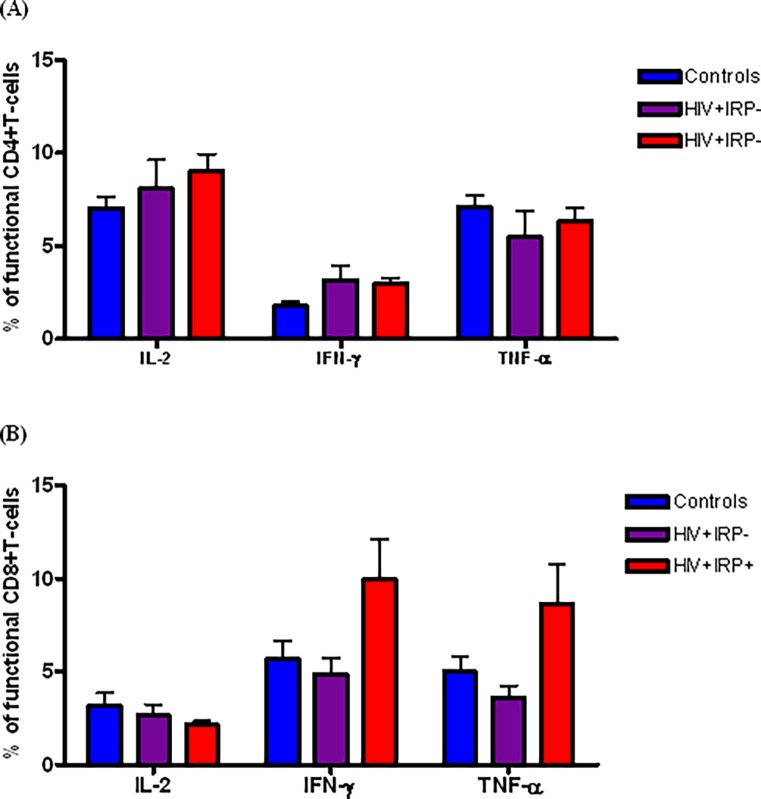
Relationship between the Immune Risk Phenotype (IRP) and Staphylococcus Enterotoxin B induced pro-inflammatory cytokine secretion. Percentages of IL-2, IFN-γ or TNF-α producing T-cells in response to Staphylococcus Enterotoxin B (SEB) stimulation within the CD4+ (A) and CD8+ (B) T-cell compartments. Comparisons were made between uninfected controls (blue), HIV+IRP+ (purple) and HIV+IRP+ (red) subjects. Statistical significance was determined using the Mann–Whitney U test. *p<0.05, **p<0.01, ***p <0.001.

Pro-inflammatory cytokines play an important role in the induction and maintenance of immune senescence [25,26]. We therefore sought to evaluate the link between the IRP and chronic inflammation by monitoring the secretion of inflammatory mediators. Following antigenic stimulation with SEB, we assessed the frequencies of CD4+ and CD8+ T-cells producing IL-2, IFN- or TNF-α. Mean frequencies of cytokine-producing CD4+T-cells in response to SEB, were similar in HIV-infected and uninfected subjects (Fig. 1A). Within the CD8+ T-cell subset, the frequency of IFN-γ producing T-cells was higher in HIV+IRP+ subjects compared with that in HIV+IRP+ and control subjects though this difference failed to achieve statistical significance (*p* = 0.07 and *p* = 0.06, respectively). The frequency of TNF-α producing CD8+ T-cells was also the highest among HIV+IRP+ subjects. Differences between this group and the HIV+IRP- group approached statistical significance (*p* = 0.05). There was no difference in IL-2 production across the three groups in either CD4+ or CD8+ T-cell compartments.

### 2. Relationship between the IRP and proliferative response

**Fig 2:**
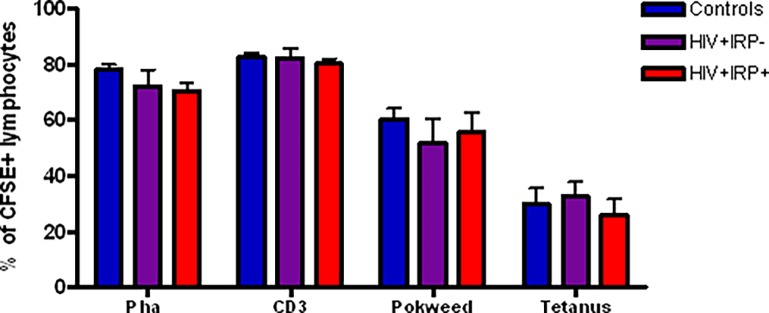
Relationship between the IRP and proliferative capacity. The percentage of carboxyfluorescein succinimidyl ester (CFSE) positive cells was used to assess the proliferative capacity of lymphocytes after stimulation with phytohemagglutinin, (PHA), anti-CD3 and-CD28, Pokeweed mitogen and Tetanus toxoid. Comparisons were made between uninfected controls (blue), HIV+IRP- (purple) and HIV+IRP+ (red). Statistical significance was determined using the Mann–Whitney U test. *p<0.05, **p<0.01, ***p <0.001.

In the uninfected elderly, the IRP has been associated with reduced *in vitro* lymphocyte proliferative capacity in response to mitogens [6]. We therefore investigated lymphocyte proliferative responses to well-defined stimuli including: PHA, anti-CD3 and-CD28 mAbs, Pokeweed mitogen and Tetanus toxoid using a CFSE dilution assay. We observed no differences in proliferation between the groups (Fig. 2). Therefore, among successfully treated HIV-infected subjects, being positive for the IRP did not influence lymphocyte proliferation responses.

### 3. Relationship between the IRP and T-cell subset distribution

**Fig 3:**
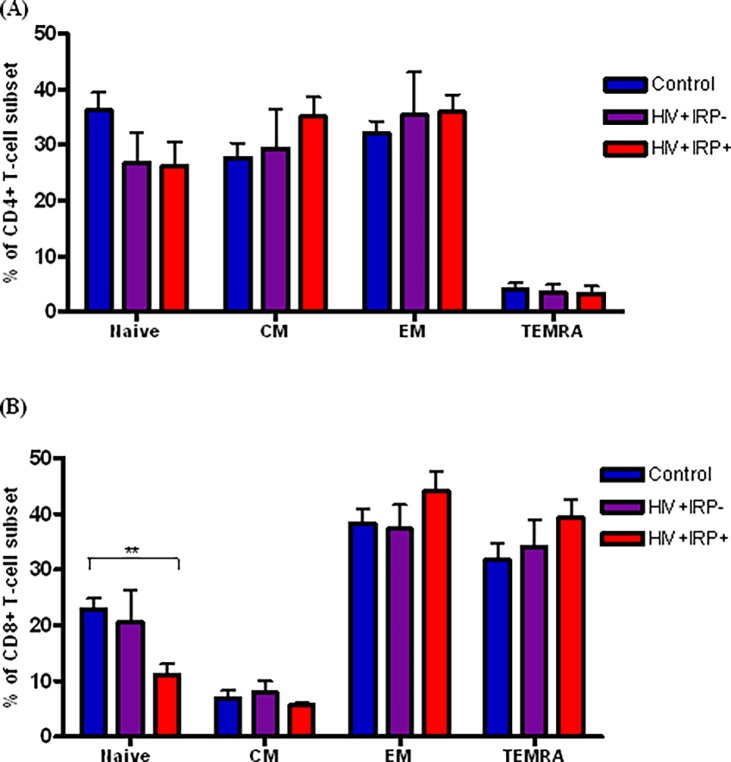
Relationship between the IRP and T-cell subset distribution. Percentages of naive (CD45RA+ CD28+CCR7+), central memory (CD45RA-CD28+CCR7+), effector memory (CD45RA- CD28-CCR7-) and terminally differentiated effector memory (TEMRA) (CD45RA+CD28- CCR7-) T-cells were assessed within the CD4+ (A) and CD8+ (B) T-cell compartments. Comparisons were made between the frequency of these subsets in uninfected controls (blue), HIV+IRP- (purple) and HIV+IRP+ (red). Statistical significance was determined using the Mann–Whitney U test. *p<0.05, **p<0.01, ***p <0.001.

CD45RA, CCR7 and CD28 are cell surface markers used to identify four phenotypically and functionally distinct subsets of CD4+ and CD8+ T-cells: naive (T_N_: CD45RA+CD28+CCR7+), central memory (T_CM_: CD45RA-CD28+CCR7+), effector memory (T_EM_: CD45RA-CD28-CCR7-) and terminally differentiated effector memory (TEMRA: CD45RA+CD28-CCR7-). Fig. 3A shows that, within the CD4+ T-cell compartment, no major between group differences in subset distribution was observed. In contrast, within the CD8+ T-cell compartment, the frequency of the T_N_ subset was significantly lower in the HIV+IRP+ than in age-matched controls (*p* = 0.007) (Fig. 3B).

### 4. Relationship between the IRP and replicative senescence

**Fig 4:**
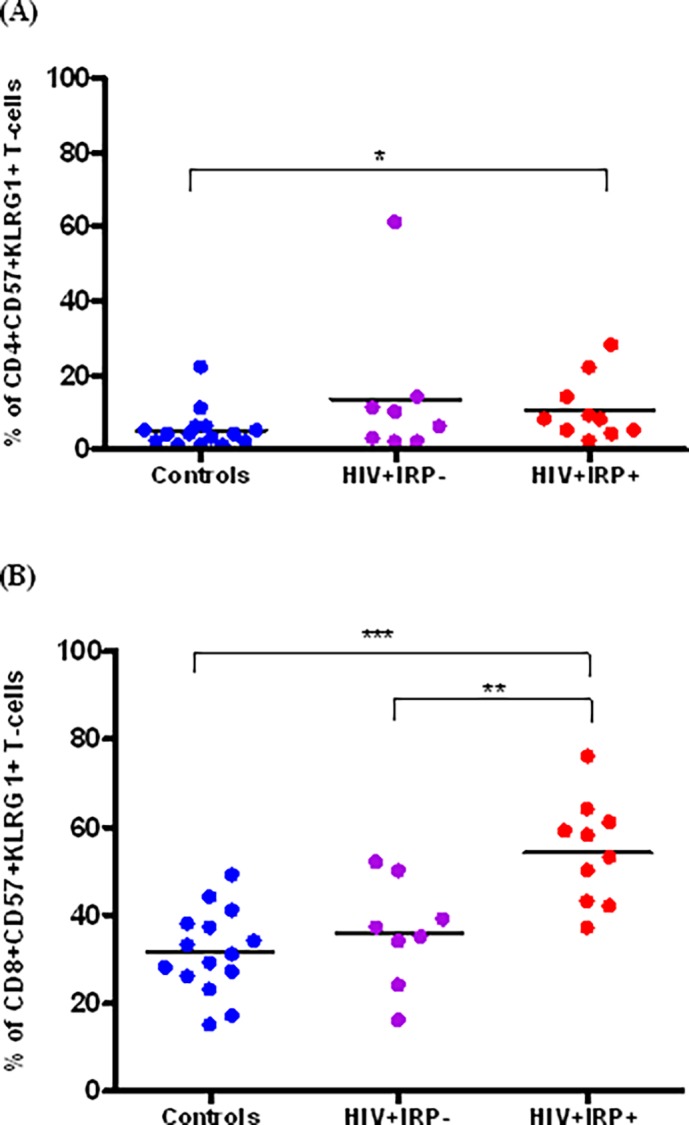
Relationship between the IRP and markers of replicative senescence. The frequency of CD4+ (A) and CD8+ (B) T-cells expressing a both CD57 and KLRG1 was measured and compared in uninfected controls (blue), HIV+IRP- (purple) and HIV+IRP+ (red) sunjects. Statistical significance was determined using the Mann–Whitney U test. *p<0.05, **p<0.01, ***p <0.001.

Increased levels of CD57 and KLRG1 expression on T-cells has been linked to immune replicative senescence and HIV-1 disease progression [27–29]. To determine whether these markers were differentially expressed in the populations studied here, we compared their expression on both CD4+ and CD8+ T-lymphocytes from HIV-infected subjects with and without IRP and with age matched controls. Within the CD4+ T-cell subset, there was no difference in the proportion of CD57+KLRG1+ T-cells between HIV+IRP+ and HIV+IRP- subjects. However, CD4+ T-cells from HIV+IRP+ subjects exhibited a significantly higher expression of senescence markers than those from age matched controls (*p* = 0.03) (Fig. 4A). Within the CD8+ T-cell compartment, the replicative senescence markers were higher on cells from HIV+IRP+ compared to HIV+IRP- and control subjects (*p* = 0.004 and 0.0003), respectively (Fig. 4B). Overall, our data indicate that the upregulation of CD57 and KLRG1 expression occurred mainly on CD8+ T-cells from HIV-infected subjects with an IRP profile.

### 5. Relationship between the IRP and lymphocyte telomere length

**Fig 5:**
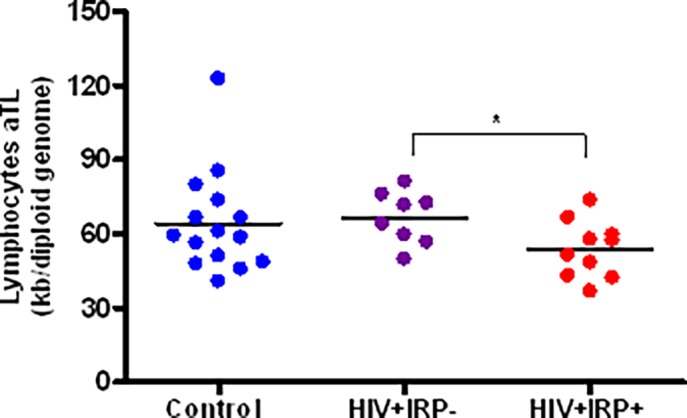
Relationship between the IRP and lymphocyte telomere length. Average telomere length (aTL) was measured in kilobases (kb) per diploid genome. Lymphocyte aTL comparisons were made between uninfected controls (blue), HIV+IRP- (purple) and HIV+IRP+ (red). Statistical significance was determined using the Mann–Whitney U test. *p<0.05, **p<0.01, ***p <0.001.

Telomere length, a well-established marker of cellular senescence and telomere attrition, has previously been associated with several age related conditions such as cardiovascular disease [30]. In order to assess the relationship between the IRP and cellular senescence, we evaluated the absolute telomere length (aTL) of DNA from lymphocytes in the three study groups. The aTL in HIV+IRP+ subjects was significantly shorter than that in HIV+IRP- (*p* = 0.03) (Fig. 5). HIV+IRP- subjects appeared to have an aTL similar to that of uninfected controls. These results indicate, for the first time, that the IRP is associated with shorter telomere length in successfully treated HIV-infected individuals.

## Discussion

The IRP is a phenotype that occurs in 15–20% of octagenarians (>85 years old) and predicts long-term morbidity and mortality in the elderly [31]. Given that IRP-specific biomarkers can also be observed in individuals with HIV, we examined the relationship between this phenotype and markers associate with immune senescence and dysregulation in the context of well-controlled HIV-infection. We found that despite effective ART, HIV-infected subjects with an IRP displayed a higher degree of immune senescence than their non-IRP counterparts.

Chronic immune inflammation plays an important role in the onset and the maintenance of immune senescence; and has been linked to non-AIDS defining illnesses (NADIs) [18,32–34]. Our findings indicated a markedly higher frequency of SEB stimulated IFN-γ and TNF-α secreting CD8+ T-cells in HIV+IRP+ subjects compared to their HIV+IRP- counterpart. Although these differences failed to achieve significance, probably due to the small sample size, this observation could suggest a potential association between the IRP and increased chronic inflammation despite well-controlled HIV infection. Given the well-established association between chronic inflammation and NADIs, the presence of the IRP might identify a subset of treated HIV-infected individuals who are at risk for non-AIDS morbidity and mortality [35,36]. Therefore these markers should be investigated in larger cohort settings.

Interestingly, our analysis did not reveal any significant differences in the proliferative capacity of lymphocyte from the three study groups. These results are in accordance with previous findings that successfully treated asymptomatic HIV-infected individuals have T-cell responses to recall antigens and mitogens that are comparable to those of healthy controls [37]. Therefore, in treated HIV-infected individuals, the IRP does not seem to be associated with proliferative dysfunction.

The depletion of naïve T-cells and the accumulation of late-stage effector memory T-cells (TEMRA) represent other key features of immune senescence that are associated with poor immune status [38,39]. Although we did not find any IRP-related increase in TEMRA, we noted a lower naïve CD8+ T-cell frequency in HIV+IRP+ vs. HIV uninfected controls. As the development of immune responses against new pathogens is dependent on the availability of naïve T-cells, these responses could be compromised in HIV+IRP+ individuals [40,41]. It will thus be important to determine whether HIV+IRP+ subjects are more likely to be susceptible to new infections or to fail to respond to vaccines involving cell-mediated immunity. Interestingly, no significant differences were observed between HIV+IRP+ subjects and HIV+IRP- or control subjects. It is possible that differences between these groups were masked by the limited sample size. Larger studies will be required to further investigate these results.

CD57 and KLRG1 are both putative markers of replicative immune senescence that are commonly used for the functional characterization of T-cells with reduced proliferative capacity, high susceptibility to apoptosis and shortened telomeres [27][29]. Previous studies have demonstrated that some CD57+ T-cells are not ‘‘truly” senescent, because they can proliferate under specific conditions [42]. On the other hand, CD57+ KLRG1+ double-positive T-cells are thought to identify a subset of terminally differentiated cells that are unable to respond to antigens [43]. Although CD4+CD57+KLRG1+ T-cells were significantly higher in HIV+IRP+ subjects than in controls, we observed no difference between the HIV+IRP- group and HIV+IRP+ or control groups. However, our data indicate a marked expansion (approximately a 1.5 fold increase) of CD8+CD57+KLRG1+ T-cells in HIV+IRP+ subjects compared to both HIV+IRP- and control subjects. The extent of replicative senescence thus appears more pronounced in HIV-infected individuals with an IRP.

As a marker of biological senescence, telomere length has been associated with the health and longevity of an individual. However, to our knowledge, this marker has never been evaluated in the context of the IRP. In this study, we demonstrated for the first time that the HIV+IRP+ subjects have shorter telomeres than HIV+IRP- subjects. Telomere length in HIV+IRP- and uninfected controls were comparable. Taken together, these data indicate that HIV+IRP+ subjects have an increased degree of cellular senescence compared to their non-IRP counterparts. This is of particular importance considering that short lymphocyte telomere length has been repeatedly associated with poor clinical outcomes in other disease states such as; diabetes, cancer and cardiovascular disease [30,44,45]. HIV+IRP+ individuals might therefore be at higher risk for aging-related co-morbidities. In fact in this small group of HIV+ individuals, we observed that none of the HIV+IRP- subjects experienced cardiovascular disease while notably 70 percent of those with an IRP had a documented cardiovascular event as defined by the occurrence of an acute coronary syndrome (myocardial infarction, diagnosed unstable angina, or stroke).

Collectively, our findings suggest that the phenotypic and functional immune characteristics of HIV+IRP+ subjects are distinct from those observed in HIV+IRP- subjects. Indeed, a greater degree of immune senescence is observed in those with an IRP, despite similar ages and viral suppression levels in the two groups. It is important to note that the individuals included in this study are much younger than the octagenarians in which the IRP was initially identified. This implies that in HIV-infected individuals, the IRP occurs at a younger age and is associated with functional and phenotypic aberrations similar to those observed in octagenarians. One of the main limitations of this study is the small sample size of our populations. Therefore, larger cohort studies are needed to validate these data. Another limitation is that the study was restricted to men, as very few women attend our clinic. Finally we were not able to include HIV uninfected controls with an IRP status, because their prevalence is very low in this age group.

In many countries, the wide availability of potent therapy has prolonged the life expectancy of HIV-infected individuals. Yet, despite long-term viral suppression some individuals are at risk for aging-related co-morbidities such as cardiovascular disease. It is thus important to understand the relationship between these comorbidities and residual immune abnormalities. In this study we identified a subset of HIV+ subjects, who, despite being virologically suppressed, exhibit immune markers associated with immune dysregulation and senescence. Given the association of each of these pathologic markers with poor health outcomes, our finding that they are prominent among HIV+IRP+ subjects may be clinically important in the context of controlled HIV infection. It is clear that, larger studies are required to investigate the predictive value of the IRP as a marker of risk in the development of aging-related co-morbidities in those with HIV.
